# Prognostic value of aberrant promoter hypermethylation of tumor-related genes in early-stage head and neck cancer

**DOI:** 10.18632/oncotarget.8317

**Published:** 2016-03-24

**Authors:** Kiyoshi Misawa, Daiki Mochizuki, Atsushi Imai, Shiori Endo, Masato Mima, Yuki Misawa, Takeharu Kanazawa, Thomas E. Carey, Hiroyuki Mineta

**Affiliations:** ^1^ Department of Otolaryngology/Head and Neck Surgery, Hamamatsu University School of Medicine, Shizuoka, Japan; ^2^ Department of Otolaryngology/Head and Neck Surgery, Jichi Medical University, Tochigi, Japan; ^3^ Department of Otolaryngology/Head and Neck Surgery, Laboratory of Head and Neck Cancer Biology, University of Michigan, Ann Arbor, MI, USA

**Keywords:** tumor-suppressor genes, hypermethylation, head and neck cancer, metastases, biomarker

## Abstract

Staging and pathological grading are useful, but imperfect predictors of recurrence in head and neck squamous cell carcinoma (HNSCC). Accordingly, molecular biomarkers that predict the risk of recurrence are necessary to improve clinical outcomes. The methylation statuses of the promoters of 11 tumor-related genes (*p16*, *RASSF1A*, *E-cadherin*, *H-cadherin*, *MGMT*, *DAPK*, *DCC*, *COL1A2*, *TAC1*, *SST*, and *GALR1*) were analyzed in 133 HNSCC cases using quantitative methylation-specific PCR. We detected frequent methylation of *p16* (44%), *RASSF1A* (18%), *E-cadherin* (53%), *H-cadherin* (35%), *MGMT* (35%), *DAPK* (53%), *DCC* (42%), *COL1A2* (44%), *TAC1* (61%), *SST* (64%), and *GALR1* (44%) in HNSCC. Disease-free survival was lower in patients with 6–11 methylated genes than in those with 0–5 methylated genes (log-rank test, *P* = 0.001). In a multivariate Cox proportional hazards analysis, the methylation of *E-cadherin, COL1A2, TAC1*, and *GALR1* was associated with poor survival, with hazard ratios of 4.474 (95% CI, 1.241–16.124). In a joint analysis of these four genes, patients with 2–4 methylated genes had a significantly lower survival rate than those with 0–1 methylated genes in early-stage HNSCC. Importantly, the methylation of some genes was closely related to poor prognosis in early-stage HNSCC, providing strong evidence that these hypermethylated genes are valuable biomarkers for prognostic evaluation.

## INTRODUCTION

The treatment strategy for patients with HNSCC is generally guided by tumor–node–metastasis (TNM) classification and clinical staging. Only 90% of stage I patients can be cured by surgery or radiotherapy. The fraction of aggressive tumors rises to 30% for stage II patients, 50% at stage III, and 70% at stage IV. These aggressive tumors relapse quickly and progress, ultimately causing death [1–3]. Biomarker development is necessary to improve our understanding of the molecular basis of HNSCC progression and to provide sufficient discriminatory prognostic power for the effective clinical management of this disease [4].

Alterations in epigenetic marks (i.e., hypermethylation events) are useful biomarkers; for example, *MGMT* epigenetic alterations are useful biomarkers in glioblastomas [5] and *GSTP1* alterations are useful in prostate cancers [6]. *MGMT* predicts the response to DNA-alkylating drugs [7]. *GSTP1* is an established biomarker for prostate cancer diagnosis and prognosis [7]. In a previous analysis of HNSCC, there was no observable effect of *p16*, *MGMT*, *DAPK*, or *E-cadherin* on prognosis for patients with laryngeal and hypopharyngeal cancer [8]. Tan et al. demonstrated that hypermethylated promoters in the surgical margins of HNSCC predict local recurrences and disease-specific deaths based on a panel of three genes (*p16, cyclin A1*, and *DCC*) [9]. According to Carvalho et al., salivary DNA promoter hypermethylation analyses facilitate the early diagnosis of HNSCC, and several hypermethylated genes (*DAPK, DCC, MINT-31, TIMP-3, p16, MGMT*, and *CCNA1*) have already been identified in salivary rinse samples [10–12]. Although several hypermethylated candidate genes have been identified in HNSCC, few are suitable methylation markers for clinical use.

Therapeutic procedures differ substantially between early- and late-stage HNSCC. Early-stage HNSCC patients receive minimally invasive surgery or irradiation alone, and late-stage patients receive aggressive therapy, such as expanded surgery and/or concomitant chemoradiotherapy [2, 3, 13]. The ability to distinguish between low- and high-risk HNSCCs at an early stage may reduce follow-up costs. We hypothesized that the quantitative methylation-specific PCR (Q-MSP) assay could be used to define patterns of DNA methylation that differentiate low- and high-risk HNSCCs.

We prospectively analyzed 11 genes involved in cell cycle control (*p16,* galanin receptor 1; *GALR1*), DNA damage repair (O^6^-alkylguanine DNA alkyltransferase; *MGMT*), apoptosis (death-associated protein kinase; *DAPK,* Ras association domain-containing protein 1; *RASSF1A*), inflammatory reactions (tachykinin, precursor 1; *TAC1*), antitumor and antisecretory activity (somatostatin; *SST*), and tumor cell invasion (*E-cadherin, H-cadherin,* deleted in colorectal carcinoma [*DCC*], and collagen alpha-2(I) chain [*COL1A2*]) in a cohort of clinically well-characterized HNSCC samples. Furthermore, we identified the most powerful combination of hypermethylated genes, and characterized an early stage-specific marker for HNSCC treatment.

## RESULTS

### Distributions of individual methylated genes

**Figure 1:**
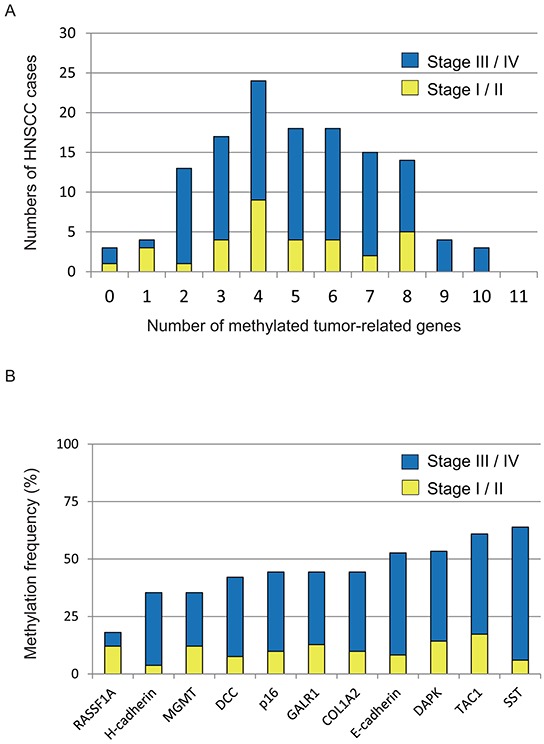
Summary of gene promoter hypermethylation in 133 HNSCC samples **A.** Bar graph showing the numbers of tumor-related methylated genes for 33 stage I/II (yellow bars) and 100 stage III/IV (blue bars) samples. **B.** Bar graph showing methylation frequencies (%) of 11 tumor-related genes in the cohort. Blue bars, samples of stage III/IV cases; yellow bars, samples of stage I/II cases.

We used Q-MSP to examine promoter methylation of 11 genes in 133 primary HNSCC tumors. One hundred and thirty primary tumors (97.7%) showed hypermethylation of at least one gene in the panel. Thirty-seven (37 of 133; 27.8%) tumors included 0 to 3 hypermethylated genes, 18.0% (24/133) had 4 hypermethylated genes, 38.3% (51/133) had 5 to 7 hypermethylated genes, and 15.8% (21/133) had 8 to 10 hypermethylated genes (Figure 1A). The mean number of methylated genes was 4.95 (range, 0–10). In particular, we detected frequent methylation of *p16* (44.4%), *RASSF1A* (18.0%), *E-cadherin* (53.6%), *H-cadherin* (35.3%), *MGMT* (35.3%), *DAPK* (53.4%), *DCC* (42.1%), *COL1A2* (44.4%), *TAC1* (61.0%), *SST* (64.0%), and *GALR1* (44.4%) in HNSCC (Figure 1B).

### Clinicopathological characteristics of primary HNSCC patients

Patient clinical features were used to examine differences in methylation index (MI) with respect to age, gender, alcohol exposure, smoking status, tumor size, lymph-node status, and stage. Based on continuous marker methylation analyses, the MI of 11 tumor-related genes (TRGs) were not correlated with any patient characteristics (Supplementary Figure S2). As summarized in Table 1, we performed a detailed analysis of methylation status for each gene according to clinical characteristics. We found that *p16* promoter methylation is inversely associated with age (Fisher's exact tests; *P* = 0.019). There was an association between methylation of the *COL1A2* promoter and gender (*P* = 0.022). Methylation of *TAC1* was significantly correlated with alcohol exposure (*P* = 0.039). Methylation of *GALR1* was significantly correlated with tumor size (*P* = 0.002) and clinical stage (*P* = 0.009). Methylation of the promoters of other genes was not associated with age at onset, gender, alcohol exposure, smoking status, tumor site, tumor size, lymph-node status, or clinical stage (Table 1).

**Table 1:** Distribution of methylation status by selected epidemiologic and clinical characteristics

Gene	Methylation status	Characteristics	Age			Gender			Alcohol exposure		
		Overall(%)	70<	70>	P ^†^	Female	Male	P ^†^	drinker	non drinker	P ^†^
p16	Yes	59 (44.4)	49	10	0.019^*^	12	47	1	38	21	1
	No	74 (55.6)	47	27		12	62		49	25	
RASSF1A	Yes	24 (18.0)	16	8	1	4	20	1	15	9	1
	No	109 (82.0)	80	29		20	89		72	37	
E-cadherin	Yes	70 (52.6)	47	23	0.182	14	56	0.653	46	24	1
	No	63 (47.4)	49	14		10	53		41	22	
H-cadherin	Yes	47 (35.3)	34	13	1	10	37	1	32	15	0.705
	No	86 (64.7)	62	24		14	72		55	31	
MGMT	Yes	47 (35.3)	30	17	1	9	38	1	30	17	1
	No	86 (64.7)	66	20		15	71		57	29	
DAPK	Yes	71 (53.4)	53	18	0.563	17	54	0.072	51	20	0.104
	No	62 (46.6)	43	19		7	55		36	26	
DCC	Yes	56 (42.1)	39	17	1	10	46	1	37	19	1
	No	77 (57.9)	57	20		14	63		50	27	
COL1A2	Yes	59 (44.4)	43	16	1	16	43	0.022*	36	23	1
	No	74 (55.6)	53	21		8	66		51	23	
TAC1	Yes	81 (60.9)	55	26	0.234	13	68	1	59	22	0.039*
	No	52 (39.1)	41	11		11	41		28	24	
SST	Yes	85 (63.9)	63	22	1	13	72	1	60	25	1
	No	48 (36.1)	33	15		11	37		27	21	
GALR1	Yes	59 (44.4)	44	15	0.7	11	48	1	36	23	1
	No	74 (55.6)	52	22		13	61		51	23	
**Smoking status**			**Tumor size**			**Lympho-node status**			**Stage**		
**smoker**	**non smoker**	**P**^†^	**T1-2**	**T3-4**	**P**^†^	**N0**	**N+**	**P**^†^	**I-II**	**III-IV**	**P**^†^
40	19	1	28	31	1	27	32	1	16	43	1
55	19		31	43		32	42		17	57	
17	7	1	12	12	1	9	15	0.503	5	19	0.795
78	31		47	62		50	59		28	81	
52	18	0.45	33	37	0.6	30	40	1	16	54	0.688
43	20		26	37		29	34		17	46	
35	12	0.689	23	24	1	18	29	0.362	10	37	0.535
60	26		36	50		41	45		23	63	
33	14	1	20	27	0.856	20	27	0.856	13	34	1
62	24		39	47		39	47		20	66	
53	18	0.443	33	38	0.605	31	40	1	17	54	1
42	20		26	36		28	34		16	46	
38	18	1	25	31	1	26	30	1	13	43	0.839
57	20		34	43		33	44		20	57	
42	17	1	23	36	0.295	22	37	0.162	11	48	0.161
53	21		36	38		37	37		22	52	
63	18	0.051	34	47	1	34	47	1	19	62	1
32	20		25	27		25	27		14	38	
61	24	1	40	45	0.469	36	49	1	23	62	0.532
34	14		19	29		23	25		10	38	
42	17	1	17	42	0.002*	22	37	0.162	8	51	0.009^*^
53	21		42	32		37	37		25	49	

† Fisher's exact probability test.

* P<0.05.

### Association between TRG methylation and survival

Table 2 illustrates the overall associations between the methylation status of individual TRGs and disease-free survival (DFS) based on a logistic regression model. After adjusting for age, gender, smoking status, stage, we found that hypermethylation of *E-cadherin, COL1A2, TAC1*, and *GALR1* was associated with significantly reduced survival, with hazard ratios of 2.263 (95% CI, 1.103–4.641), 3.824 (95% CI, 1.794–8.152), 3.216 (95% CI, 1.491–6.937), and 3.125 (95% CI, 1.489–6.557), respectively (Table 2).

**Table 2:** Methylation status of individual genes and associations with disease-free survival using Logistic regression model

Gene	Methylation status	Overall(%)	Recurrence events		Adjusted RR (95% CI)^†^
			Positive (N = 67)	Negative (N = 66)	
p16	Yes	59 (44.4)	32	27	1.300 (0.632-2.672)
	No	74 (55.6)	35	39	
RASSF1A	Yes	24 (18.0)	13	11	1.214 (0.490-3.009)
	No	109 (82.0)	54	55	
E-cadherin	Yes	70 (52.6)	42	28	2.311 (1.126-4.744)^*^
	No	63 (47.4)	25	38	
H-cadherin	Yes	47 (35.3)	29	18	1.961 (0.935-4.113)
	No	86 (64.7)	38	48	
MGMT	Yes	47 (35.3)	27	20	1.639 (0.780-3.444)
	No	86 (64.7)	40	46	
DAPK	Yes	71 (53.4)	41	30	1.751 (0.857-3.580)
	No	62 (46.6)	26	36	
DCC	Yes	56 (42.1)	31	25	1.419 (0.701-2.875)
	No	77 (57.9)	36	41	
COL1A2	Yes	59 (44.4)	41	18	3.824 (1.802-8.117)*
	No	74 (55.6)	26	48	
TAC1	Yes	81 (60.9)	49	32	3.824 (1.802-7.532)*
	No	52 (39.1)	18	34	
SST	Yes	85 (63.9)	45	40	1.485 (0.712-3.099)
	No	48 (36.1)	22	26	
GALR1	Yes	59 (44.4)	39	20	3.132 (1.489-6.585)*
	No	74 (55.6)	28	46	

† Adjusted for age (70 and older vs. <70), gender, smoking status and stage (I, II vs. III, IV).

* P<0.05.

**Figure 2:**
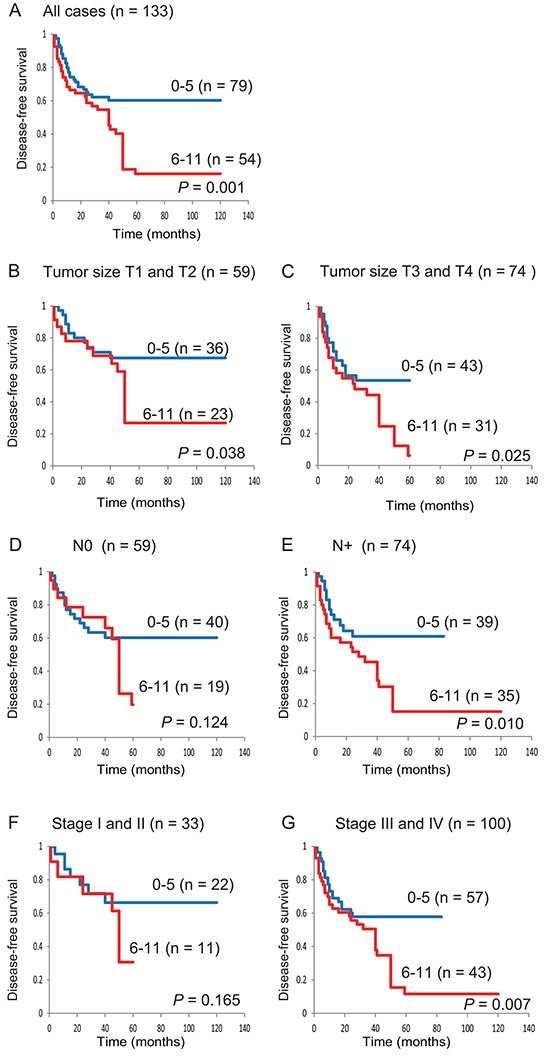
Kaplan-Meier survival curves for patients with HNSCC according to the methylation status of 11 tumor-related genes Disease-free survival for **A.** all 133 HNSCC cases, **B.** tumor size T1 and T2 cases (n = 59), **C.** tumor size T3 and T4 cases (n = 74), **D.** lymph-node status N0 cases (n = 59), **E.** lymph-node status N+ cases (n = 74), **F.** stage I and II cases (n = 33), and **G.** stage III and IV cases (n = 100). Blue line: patients with 0–5 methylated genes; red line: patients with 6–11 methylated genes.

Based on log-rank tests, we detected an association between poor survival and the methylation phenotype defined as ≥6 methylated genes (*P* = 0.001) (Supplementary Table S1). Kaplan-Meier plots indicated that methylation of 11 TRGs in patient samples was related to the duration of DFS. The DFS was lower in patients with 6–11 methylated genes than in the group with 0–5 methylated genes (60.3% versus 16.1%, respectively; log-rank test, *P* = 0.001) (Figure 2A). Among 59 patients with T1 and T2 tumors, the DFS rate was 26.8% in the group of patients with 6–11 methylated genes and was 67.5% in the 0–5 group (log-rank test, *P* = 0.038) (Figure 2B). Among 59 patients with N0 lympho-node status, there was no significant association between patients with 6–11 methylated genes and 0 to 5 methylated genes (log-rank test, *P* = 0.124) (Figure 2D). Among 33 patients with stage I and II patients, no correlation was found between patients with a high (6 to 11) and low (0 to 5) number of methylated genes (log-rank test, *P* = 0.165) (Figure 2F). In 100 stage III and IV patients, the DFS was statistically significantly worse in the group with a high number of methylated genes (log-rank test, *P* = 0.007) (Figure 2G).

**Figure 3:**
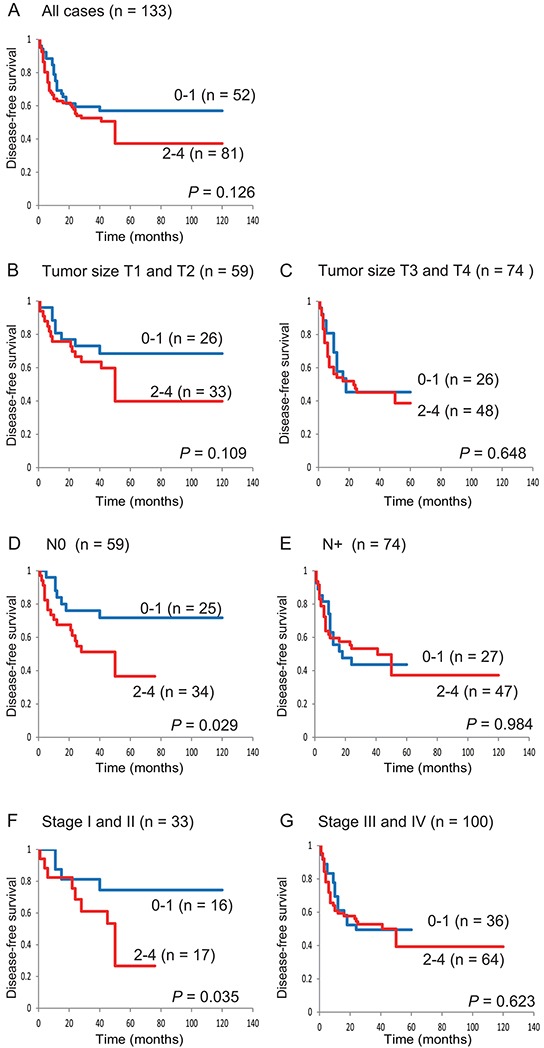
Kaplan-Meier survival curves for patients with HNSCC according to *E-cadherin*, *COL1A2*, *TAC1*, and *GALR1* methylation status Disease-free survival for **A.** all 133 HNSCC cases, **B.** tumor size T1 and T2 cases (n = 59), **C.** tumor size T3 and T4 cases (n = 74), **D.** lymph-node status N0 cases (n = 59), **E.** lymph-node status N+ cases (n = 74), **F.** stage I and II cases (n = 33), and **G.** stage III and IV cases (n = 100). Blue line: patients with 0–1 methylated genes; red line: patients with 2–4 methylated genes.

Patients with 2 to 4 methylated genes (in an analysis of *E-cadherin, COL1A2, TAC1*, and *GALR1*) had a trend toward worse survival than those with 0 to 1 methylated genes (57.0% versus 37.3%, respectively; log-rank test, *P* = 0.126) (Figure 3A). For the 4 genes, among 59 patients with T1 and T2 tumor sizes, DFS was slightly lower in the 2–4 methylated genes group than in the 0–1 methylated genes group (39.8% versus 68.5%, respectively; log-rank test, *P* = 0.109) (Figure 3B). Among 59 patients with N0 lymph-node status, patients with 0 to 1 methylated genes showed significantly better DFS than patients with 2 to 4 methylated genes (71.8% versus 36.6%, respectively; log-rank test, *P* = 0.029) (Figure 3D). Among 33 stage I and II patients, those with 0 to 1 methylated genes showed significantly better DFS than patients with 2 to 4 hypermethylated genes (74.5% versus 26.7%, respectively; log-rank test, *P* = 0.035) (Figure 3F). These data indicate that the methylation profiles of *E-cadherin, COL1A2, TAC1*, and *GALR1* are a powerful combination for the prediction of early-stage HNSCC.

Moreover, based on a multivariate Cox proportional hazard regression of 59 patients with N0 lymph-node status, which included age, gender, smoking status, and tumor stage, the group with methylation of *E-cadherin, COL1A2, TAC1,* and *GALR1* had a 4.474-times greater hazard than the group without methylation (Table 3).

**Table 3:** The promoter hypermethylation pattern and associations with disease-free survival using Cox proportional hazards model in 59 patients with N0 lymph-node status

Gene (Any)	Methylation status	Overall(%)	Recurrence events		Adjusted HR (95% CI)^†^
			Positive(N=28)	Negative(N=31)	
E-cadherin & COL1A2	Yes	41 (69.5)	23	18	2.359 (0.989-5.629)
	No	18 (30.5)	5	13	
E-cadherin & TAC1	Yes	44 (74.6)	25	19	1.989 (0.833-4.748)
	No	15 (25.4)	3	12	
E-cadherin & GALR1	Yes	36 (61.0)	20	16	2.266 (0.994-5.170)
	No	23 (39.0)	8	15	
COL1A2 & TAC1	Yes	41 (69.5)	24	17	1.202 (0.535-2.701)
	No	18 (30.5)	4	14	
COL1A2 & GALR1	Yes	32 (54.2)	19	13	5.097 (1.597-16.266)*
	No	27 (45.8)	9	18	
TAC1 & GALR1	Yes	43 (72.9)	24	19	2.170 (0.910-5.172)
	No	16 (27.1)	4	12	
E-cadherin & COL1A2 & TAC1	Yes	50 (84.7)	27	23	1.704 (0.681-4.261)
	No	9 (15.3)	1	8	
E-cadherin & COL1A2 & GALR1	Yes	43 (72.9)	23	20	3.288 (1.286-8.407)*
	No	16 (27.1)	5	11	
E-cadherin & TAC1 & GALR1	Yes	47 (79.7)	26	21	2.753 (1.048-7.229)*
	No	12 (20.3)	2	10	
COL1A2 & TAC1 & GALR1	Yes	46 (78.0)	24	22	2.782 (0.997-7.766)*
	No	13 (22.0)	4	9	
E-cadherin & COL1A2 & TAC1 & GALR1	Yes	50 (84.7)	26	24	4.474 (1.241-16.124)*
	No	9 (15.3)	2	7	

† Adjusted for age (70 and older vs. <70), gender, smoking status and stage (I, II vs. III, IV).

* P<0.05.

## DISCUSSION

We found that aberrant patterns of promoter methylation in primary tumors are indicators of an increased risk of recurrence in patients with stage I and II HNSCC. Patients with clinical stage I and II (T1-2N0) oral squamous cell carcinoma usually undergo partial glossectomy alone. However, approximately 25% of these patients develop delayed neck metastasis, which may lead to an unfavorable course [14]. Similarly, the outcomes of patients with T1-T2N0 larynx and hypopharynx cancer who are initially treated with radiotherapy or minimally invasive surgery followed by conservative surgery for radiation failure are unclear [15]. Biomarker discovery for early-stage HNSCC is crucial to improve patient outcomes. Using surgical tissues from a pilot cohort of 33 stage I/II HNSCC patients, we identified markers that may be suitable prognostic indicators for local recurrence and poor survival.

The methylation of *E-cadherin, COL1A2, TAC1,* and *GALR1* in primary early-stage HNSCC indicated metastatic risk in regional lymph nodes and distant organs. The methylation of *p16* and *H-cadherin* is associated with the early recurrence of stage I non-small cell lung carcinoma [4]. We speculate that the detection of early hypermethylation events in HNSCC tumors diagnosed as tumor-free by conventional imaging analysis can be used to identify subjects at risk of recurrence.

*E-cadherin* promoter methylation has been detected in many tumor types [16]. Low *E-cadherin* expression is associated with an increased risk of late cervical metastasis in stage I and II oral cancer patients [17], and the overexpression of *SIP1* and downregulation of *E-cadherin* predict delayed neck metastasis in stage I and II oral tongue carcinoma after partial glossectomy [18]. However, the observed methylation levels of *E-cadherin* vary among studies with respect to cancer type and survival.

*COL1A2* is a fibrillar collagen found in most connective tissues, and is the main component of the organic part of bones. *COL1A2* inactivation contributes to increased proliferation and migration activity of bladder cancer and osteosarcoma cells [19, 20]. Aberrant *COL1A2* promoter methylation has been detected in various cancer types, such as breast carcinoma [21], medulloblastoma [22], and melanoma [23, 24]. Our data also suggested that hypermethylation of *COL1A2* is associated with improved survival in patients with HNSCC [25].

*TAC1* encodes substance P, which has proliferative and anti-apoptotic effects via the activation of the ERK1/2 and nuclear factor-κB pathways, and neurokinin A, which has antiproliferative properties [3]. Hypermethylation of *TAC1* has been described in esophageal [26], gastric [27], colon [28], and breast cancer [29]. DFS is correlated with *TAC1* methylation (log-rank test, *P* = 0.002), but not with *TACR1* methylation in HNSCC [30].

GALR1 inhibits HNSCC cell proliferation via ERK1/2-mediated effects on cell cycle control proteins, such as p27, p57, and cyclin D1 [31]. We also found that *GALR1* methylation is associated with a significantly worse survival rate in HNSCC patients [32]. Doufekas et al. have recently shown that *GALR1* methylation in vaginal swabs from women with postmenopausal bleeding indicates endometrial malignancy with high sensitivity and specificity [33]. Guo et al. demonstrated that the methylation profiles of *GALR1, AGTR1, SLC5A8, ZMYND10*, and *NTSR1* are a powerful combination for non-small cell lung cancer prediction [34].

Accordingly, many genes have been reported as individual biomarkers for prognosis in HNSCC. However, combined hypermethylation patterns increased predictive power for early-stage HNSCC. The current method to assess the risk of recurrence in patients with early-stage HNSCC is imprecise; half of such tumors recur after curative surgery. The correlation between short survival times and the number of methylated genes in the regional lymph nodes supports the presence of micrometastases at those sites. We identified a combination of hypermethylated genes that increase the predictive ability for early-stage HNSCC. Simultaneous analyses of the methylation status of multiple tumor suppressor genes are important for predictions of tumorigenesis, biological behavior, and the development of future targeted therapy.

In conclusion, the methylation profiles of *E-cadherin, COL1A2, TAC1*, and *GALR1* were the most powerful combination for predicting early-stage HNSCC. This demonstrates that molecular stratification may predict cancer progression. These findings can benefit HNSCC screening and surveillance algorithms. Although our study was retrospective, was conducted at a single institution, and the number of patients was small, it serves as a platform to establish optimal therapeutic strategies for early-stage HNSCC.

## MATERIALS AND METHODS

### Tumor samples

A total of 133 primary HNSCC samples in an original cohort were obtained during surgery in the Department of Otolaryngology–Head and Neck Surgery, Hamamatsu University School of Medicine between 1977 and 2011. Clinical information, including age, sex, tumor site, smoking status, alcohol exposure, tumor size, lymph node status, stage, and recurrence events were obtained from clinical records. The male to female ratio was 109:24, and the mean age was 64.1 years (range 39–90). The primary tumors were located in the oral cavity (n = 45), hypopharynx (n = 31), larynx (n = 25), oropharynx (n = 22), and paranasal cavity (n = 10). The patients were stage I/II (n = 33) and stage III/IV (n = 100). Thirty-six matched pairs of head and neck tumor tissues and adjacent normal mucosal tissues were obtained from the surgical specimens.

### Bisulfite modification and quantitative methylation-specific PCR analysis

Genomic DNA was obtained from tumor and normal mucosal tissues using the QIAamp DNA Mini Kit (Qiagen, Courtaboeuf, France). DNA was subjected to bisulfite treatment, as described previously. [32] The bisulfite-modified DNA was used as a template for fluorescence-based real-time PCR. [35] The amplifications were performed using the TaKaRa Thermal Cycler Dice™ Real Time System TP800 (TaKaRa, Tokyo, Japan). The Q-MSP primers for methylated DNA were Q-MSP-*ACTB*-F (5′-TGGTGATGGAGGAGGTTTAGAAGT-3′) and Q-MSP-*ACTB*-R (5′-AACCAATAAAACCTACTCCTCCCTTAA-3′). A standard curve was generated using serial dilutions of universal methylated DNAs (EpiScope™ Methylated HCT116 gDNA; TaKaRa, Tokyo, Japan). The normalized methylation value (NMV) was defined as follows: NMV = *(TRGs-S/TRGs-FM)/(ACTB-S/ACTB-FM*), where *TRGs-S* and *TRGs-FM* represent TRG methylation levels in the sample and universally methylated DNAs, respectively, and *ACTB-S* and *ACTB-FM* correspond to b-actin in the sample and universally methylated DNAs, respectively. To analyze the methylation status of *p16* [36], *RASSF1A* [36], *CDH1* [36], *CDH13* [37], *MGMT* [38], *DAPK* [38], *DCC* [39], *COL1A2* [25], *TAC1* [30], *SST* [40], and *GALR1* [32], primers and conditions were used as previously described.

### Data analysis and statistics

A receiver-operator characteristic (ROC) curve analysis was performed using the NMVs for the 36 HNSCC and 36 adjacent normal mucosal tissues by StatMate IV (ATMS Co., Ltd., Tokyo, Japan). Using this approach, the area under the ROC curve indicated the optimal sensitivity and specificity levels at which to distinguish normal tissues from HNSCC tissues, and NMV thresholds were calculated for the TRGs. The cutoff value determined from this ROC curve was applied to determine TRG methylation frequency (Supplementary Figure S1). To determine the overall methylation rate in individual samples, the methylation index (MI) was used. [41, 42] The MI for each sample was defined as the ratio of the number of methylated genes to the number of genes tested (i.e., 11). The selected MI was defined as the number of methylated genes relative to the total number of genes tested (for *E-cadherin, COL1A2, TAC1*, and *GALR1*).

For the frequency analysis in the contingency tables, the associations between variables and methylation status were analyzed statistically using Fisher's exact tests. The disease-free interval was measured from the date of treatment to the date when locoregional recurrence or distant metastasis was diagnosed. DFS probabilities were estimated by the Kaplan-Meier method, and the log-rank test was applied to assess the significance of differences among actuarial survival curves. Multivariate logistic-regression analysis considering age (70 and older vs. <70), sex, smoking status, stage (I, II vs. III, IV), and methylated genes was used to identify the predictive value of the prognostic factors. Cox's proportional hazards regression analysis, which included age (70 and older vs. less than 70), sex, smoking status, stage (I, II vs. III, IV), and any methylated genes, was used to identify the multivariate predictive value of the prognostic factors [43, 44]. A significant difference was identified when the probability was less than 0.05. Statistical analyses were implemented in StatMate IV (ATMS Co., Ltd., Tokyo, Japan).

## SUPPLEMENTARY FIGURES AND TABLES


