# Supplementary material for: Prognostic value of aberrant promoter hypermethylation of tumor-related genes in early-stage head and neck cancer
Source: Oncotarget. 2016 Mar 24;7(18):26087–98. doi: 10.18632/oncotarget.8317 (PMC5041966; doi:10.18632/oncotarget.8317)
Supplement: Supplementary file 1 [file oncotarget-07-26087-s001.pdf]

SUPPLEMENTARY FIGURES AND TABLES

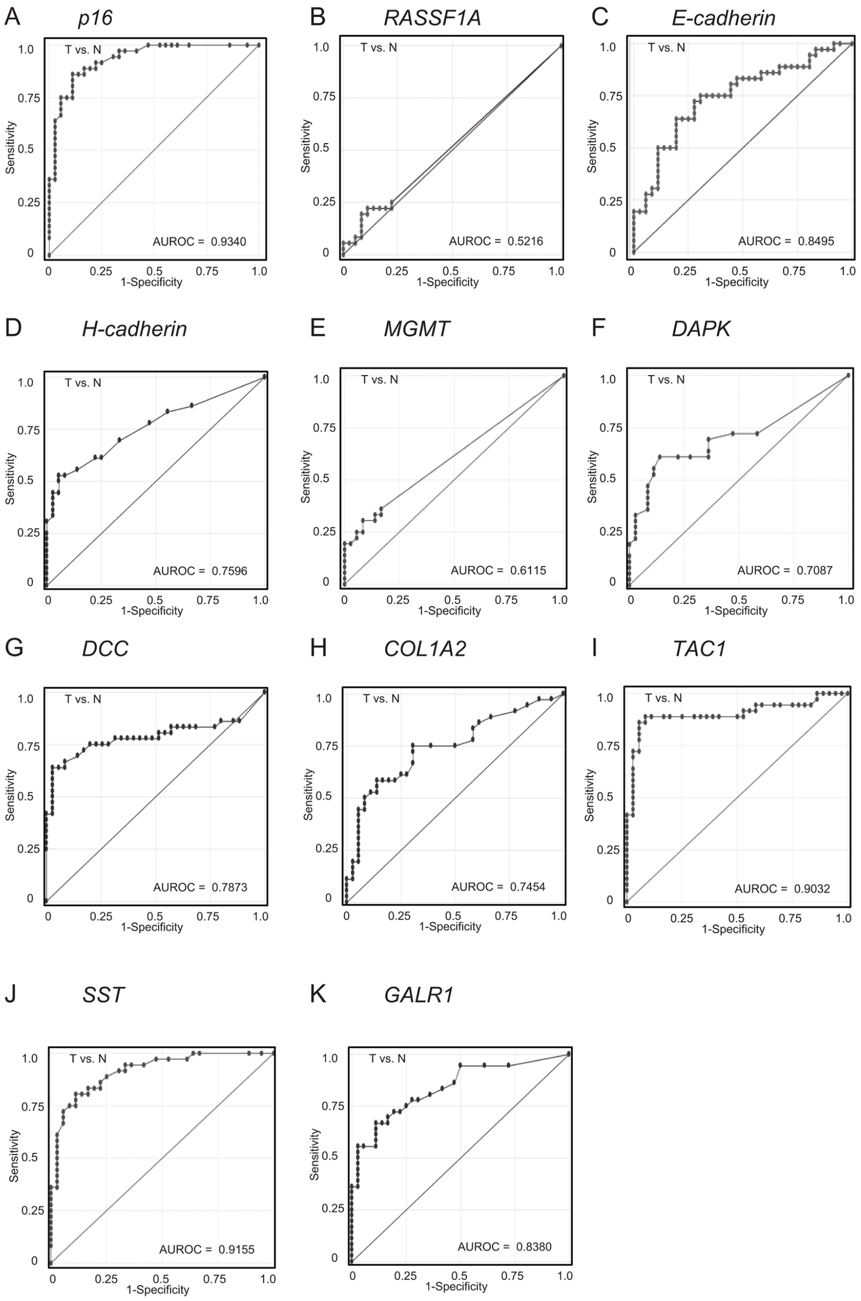

Supplementary Figure S1: Receiver Operating Characteristic (ROC) curves for individual methylation markers in HNSCC tissues (T) vs. adjacent normal mucosal tissues (N). A. Based on the ROC curve analysis, the sensitivity, specificity, and cutoff were 86.1%, 88.9%, and 0.038, respectively, for p16; **B.** 22.2%, 88.9%, and 0.040, respectively, for RASSF1A; **C.** 38.9%, 88.9%, and 0.140, respectively, for E-cadherin; **D.** 52.8%, 94.4%, and 0.090, respectively, for H-cadherin; **E.** 30.6%, 91.7%, and 0.004, respectively, for MGMT; **F.**61.1%, 86.1%, and 0.009, respectively, for DAPK; **G.** 63.9%, 97.1%, and 0.100, respectively, for DCC; **H.** 75.0%, 69.4%, and 0.012, respectively, for COL1A2; **I.** 72.2%, 97.2%, and 0.108, respectively, for TAC1; **J.** 80.6%, 88.9%, and 0.041, respectively, for SST; **K.** and 50.0%, 97.2%, and 0.038, respectively, for GALR1.

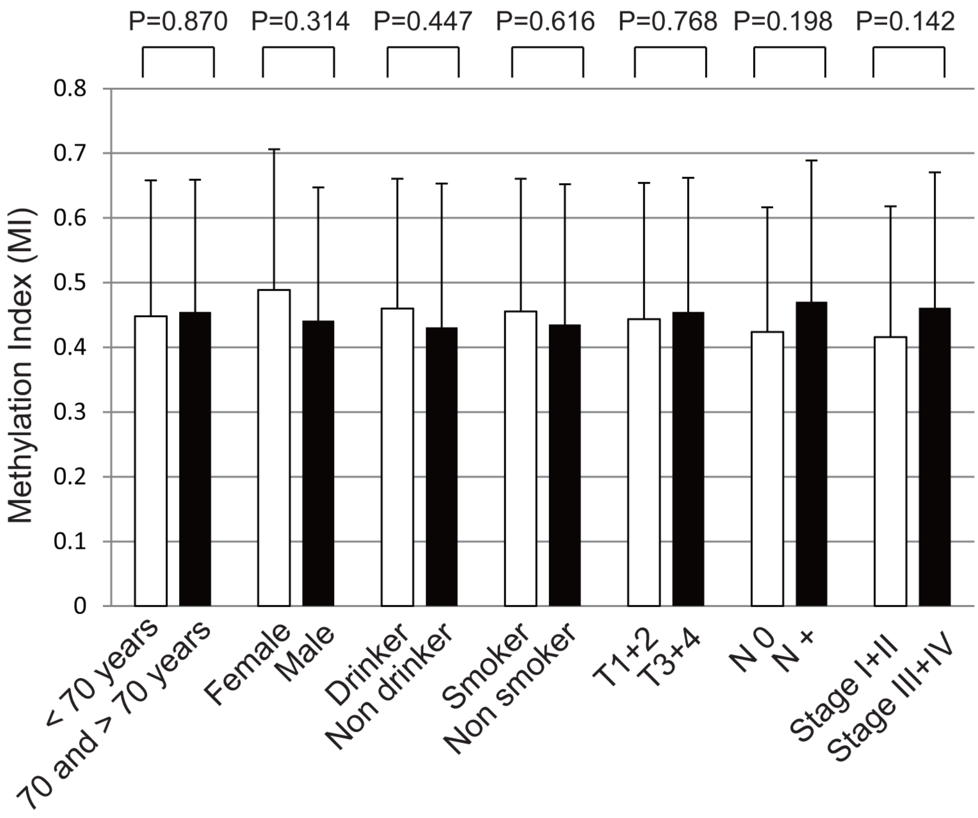

Supplementary Figure S2: Comparative analysis of methylation indexes (MI) with respect to selected clinical parameters. The mean MI for different groups was compared using Student's *t*-tests.

Supplemental Table S1: Results of log-rank tests for effect of number of methylated genes on disease free survival in 133 HNSCC

No. methylated genes	No. patients with profile	P
≥0	133	
≥1	130	0.129
≥2	126	0.073
≥3	113	0.102
≥4	96	0.015*
≥5	72	0.020*
≥6	54	0.001*
≥7	36	0.022*
≥8	21	0.113
≥9	7	0.014*
≥10	3	0.046*

Supplemental Table S2: The promoter hypermethylation pattern and associations with disease-free survival using Logistic regression model

Gene	Methylation status	Overall(%)	Recurrence events		Adjusted RR (95% CI)†
			Positive (N=67)	Negative (N=66)	
E-cadherin & COL1A2	Yes	35 (26.3)	26	9	
	No	98 (73.7)	41	57	3.700 (1.528-8.955)*
E-cadherin & TAC1	Yes	52 (39.1)	35	17	
	No	81 (60.9)	32	49	3.465 (1.598-7.512)*
E-cadherin & GALR1	Yes	41 (30.8)	31	10	
	No	92 (69.2)	36	56	4.771 (2.022-11.260)*
COL1A2 & TAC1	Yes	39 (29.3)	30	9	
	No	94 (70.7)	37	57	5.380 (2.228-12.991)*
COL1A2 & GALR1	Yes	37 (27.8)	29	8	
	No	96 (72.2)	38	58	5.097 (2.067-12.566)*
TAC1 & GALR1	Yes	40 (30.1)	31	9	
	No	93 (69.9)	36	57	6.007 (2.451-14.721)*
E-cadherin & COL1A2 & TAC1	Yes	28 (21.1)	22	6	
	No	105 (78.9)	45	60	4.752 (1.735-13.011)*
E-cadherin & COL1A2 & GALR1	Yes	24 (18.0)	20	4	
	No	109 (82.0)	47	62	5.798 (1.821-18.459)*
E-cadherin & TAC1 & GALR1	Yes	30 (22.6)	25	5	
	No	103 (77.4)	42	61	7.166 (2.469-20.797)*

(Continued)

Gene	Methylation status	Overall(%)	Recurrence events		Adjusted RR (95% CI)†
			Positive (N=67)	Negative (N=66)	
COL1A2 & TAC1 & GALR1	Yes	28 (21.1)	23	5	
	No	105 (78.9)	44	61	6.282 (2.162-18.254)*
E-cadherin & COL1A2 & TAC1 & GALR1 (Full)	Yes	22 (16.5)	18	4	
	No	111 (83.5)	49	62	5.087 (1.574-16.435)*

† Adjusted for age, gender, smoking status and stage.
* P<0.05.
